# Identification of an operon involved in fluoride resistance in *Enterobacter cloacae* FRM

**DOI:** 10.1038/s41598-017-06988-1

**Published:** 2017-07-28

**Authors:** Xiaoqing Liu, Jian Tian, Lihui Liu, Tao Zhu, Xiaoxia Yu, Xiaoyu Chu, Bin Yao, Ningfeng Wu, Yunliu Fan

**Affiliations:** 1 Biotechnology Research Institute, Chinese Academy of Agricultural Sciences, Beijing, 100081 China; 2 Key Laboratory for Feed Biotechnology of the Ministry of Agriculture, Feed Research Institute, Chinese Academy of Agricultural Sciences, Beijing, 100081 China

## Abstract

Fluorine is ubiquitous and the most active non-metal element in nature. While many microorganisms have developed fluoride resistance as a result of the widespread and prolonged application of oral hygiene products, the mechanisms used by these organisms to overcome fluoride toxicity are incompletely understood. In this study, a fluoride-resistant strain, *Enterobacter cloacae* FRM, was identified which could grow well at a fluoride concentration of 4,000 mg/L. According to comparative genomics, transcriptome under fluoride stress, and sequence analyses of two fluoride-resistant fosmid clones, the genomic island GI3 was found to be important for fluoride resistance. The result of quantitative RT-PCR indicated that six genes on GI3, *ppaC*, *uspA*, *eno*, *gpmA*, *crcB*, and orf5249, which encode a fluoride transporter, fluoride-inhibited enzymes, and a universal stress protein, reside in an operon and are transcribed into two mRNAs activated by fluoride with a fluoride riboswitch. The results of knockout and complementation experiments indicated that these genes work together to provide high fluoride resistance to *E. cloacae* FRM. This study clarified the resistance mechanism of this high fluoride-resistant organism and has expanded our understanding of the biological effects of fluoride.

## Introduction

Fluoride is ubiquitous in nature and is commonly used as an additive in water and oral hygiene products because of its strong anticaries function^1, 2^. At high concentration, fluoride exhibits toxicity against bacteria, fungi, plants, and animals. Many microorganisms have developed resistance to fluoride as a result of its widespread and prolonged application^3, 4^. However, the exact mechanisms used by fluoride-resistant microorganisms to overcome the toxicity are incompletely understood.

Currently, fluoride resistance is thought to be mediated by sensor and mitigation systems, which have been explained previously^5^. This research group found that the conserved RNA structure identified by bioinformatics in many species of bacteria and archaea^6^ functions as a fluoride-sensing riboswitch. These fluoride riboswitches regulate the expression of downstream genes to alleviate the deleterious effects of fluoride. Two of the most common genes associated with fluoride riboswitches are *crcB* and *eriC* ^*F*^. The *crcB* gene of *Escherichia coli* and the *eriC* ^*F*^ gene of *Pseudomonas syringae* function as fluoride transporters^5^. Additional studies have also implicated the proteins CrcB and EriC^F^ in fluoride resistance. CLC^F^ has been shown to function as a fluoride channel, exporting fluoride to protect *E. coli* against its toxicity^7^. Fluoride-resistant *Streptococcus mutans*, a causative agent of dental caries, which appears to lack a fluoride riboswitch^8^ encodes two homologs of the EriC^F^ protein. A single-nucleotide mutation was identified in the promoter of these two *eriC* ^*F*^ genes^9^ and this mutation was found to confer fluoride resistance on *S. mutans* by increasing promoter activity and upregulating the expression of downstream fluoride antiporter-coding genes^10^. Moreover, these *eriC* ^*F*^ genes were confirmed to confer fluoride resistance in *S. mutans* ^11^. It has also been shown that either EriC^F^ or CrcB plays a role in fluoride resistance in oral *Streptococci* ^12^. In these studies, the highest resistance to fluoride was observed at 600 mg/L.

Our laboratory has isolated a bacterium, *Enterobacter cloacae* FRM with very high fluoride resistance. Although environmental levels of fluoride are typically found in the 10–100 μM range, this bacterium can grow at 4,000 mg/L (210 mM) fluoride. Given that such high fluoride resistance is not common among fluoride-resistant microorganisms, we decided to investigate the mechanism of resistance. To this aim, the genome of the strain was sequenced, the transcriptome under fluoride conditions was analyzed by RNA sequencing, and a fosmid library was constructed to survey fluoride resistance-related genes. The expression characteristics of these genes were analyzed, and their contributions to fluoride resistance were confirmed with a series of knockout and complementation experiments. An operon containing six genes (*ppaC*, *uspA*, *eno*, *gpmA*, *crcB*, and orf5249) in the genomic island GI3 was identified and shown to be important for the survival of *E*. *cloacae* FRM in high concentration of fluoride.

## Results

### *E*. *cloacae* FRM exhibits high fluoride resistance

A fluoride-tolerant bacterial strain was isolated in our laboratory. 16S rDNA sequence analysis revealed that this strain belongs to the genus *Enterobacter*, showing the closest similarity to the *Enterobacter* strains. Based on nucleotide homology, this strain was assigned as *Enterobacter cloacae* FRM (GenBank Accession Number: CP019889, CP019890, and CP019891), with 100% identity with its nearest homolog species, *E*. *cloacae* ECNIH2 (GenBank Accession Number: CP008823).

**Figure 1:**
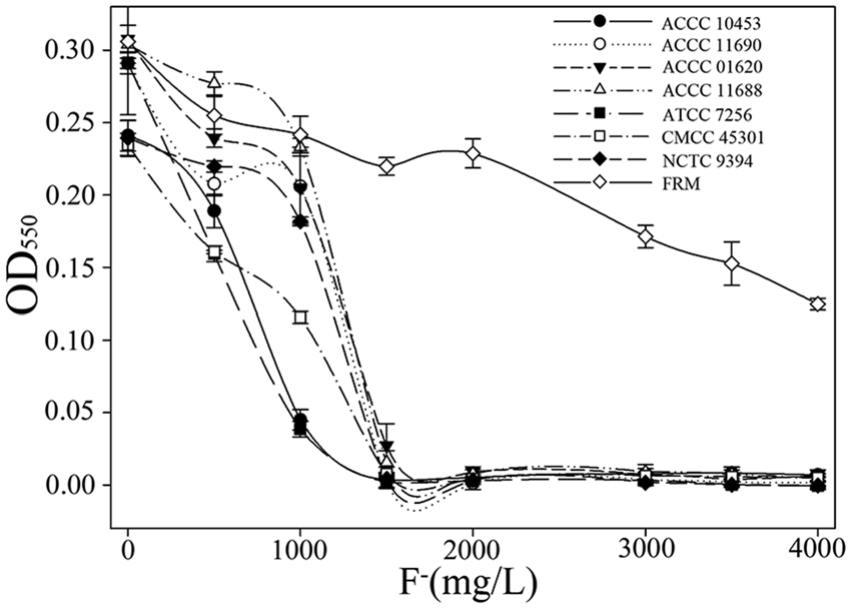
Growth of *Enterobacter cloacae* strains (FRM, ACCC 10453, ACCC 11690, ACCC0 1620, ACCC 11688, ATCC 7256, CMCC 45301, and NCTC 9394) at varying concentrations of fluoride.

To determine the level of fluoride resistance of *E*. *cloacae* FRM, we examined the growth of *E*. *cloacae* FRM, as well as other seven *E*. *cloacae* strains (*E*. *cloacae* ACCC 10453, ACCC 11690, ACCC0 1620, ACCC 11688, ATCC 7256, CMCC 45301, and NCTC 9394) in liquid medium with various fluoride concentrations. *E*. *cloacae* FRM could be grown in the presence of fluoride at concentrations up to 4,000 mg/L (Fig. 1), while the growth of the seven other *E*. *cloacae* strains was inhibited in the presence of 1,500 mg/L fluoride (Fig. 1). These data indicated that *E*. *cloacae* FRM exhibited greater fluoride resistance than many other *E*. *cloacae* strains.

### Genomic analysis of *E*. *cloacae* FRM

To elucidate the mechanism of fluoride resistance of *E*. *cloacae* FRM, the genome of *E*. *cloacae* FRM was sequenced and found to consist of a single chromosome (4,899,400 bp and 4,661 genes) and two plasmids, designated as p1 (124,734 bp and 160 genes) and p2 (112,186 bp and 135 genes). Three genomic islands were also identified within the chromosome with IslandViewer 3 (GI1, GI2, and GI3) (Fig. S1).

Ortholog analysis of the genome of *E*. *cloacae* FRM and the seven other *Enterobacteriaceae* strains identified 527 orthologous genes with the RSD algorithm. Based on these genes, a phylogenetic tree was constructed with Phylip software (Fig. S2), which revealed that the closest relative of *E*. *cloacae* FRM is *E*. *cloacae* NCTC 9394. Comparative analysis of the genome sequences of *E*. *cloacae* FRM and *E*. *cloacae* NCTC 9394 indicated that the genome of *E*. *cloacae* NCTC 9394 did not contain the three genomic islands or the plasmids. As the fluoride resistance of *E*. *cloacae* NCTC 9394 was very low (Fig. 1), we hypothesized that the genes involved in fluoride resistance may be located on the genomic islands or plasmids.

### The third genomic island, GI3, is important for the fluoride resistance of *E*. *cloacae* FRM

**Figure 2:**
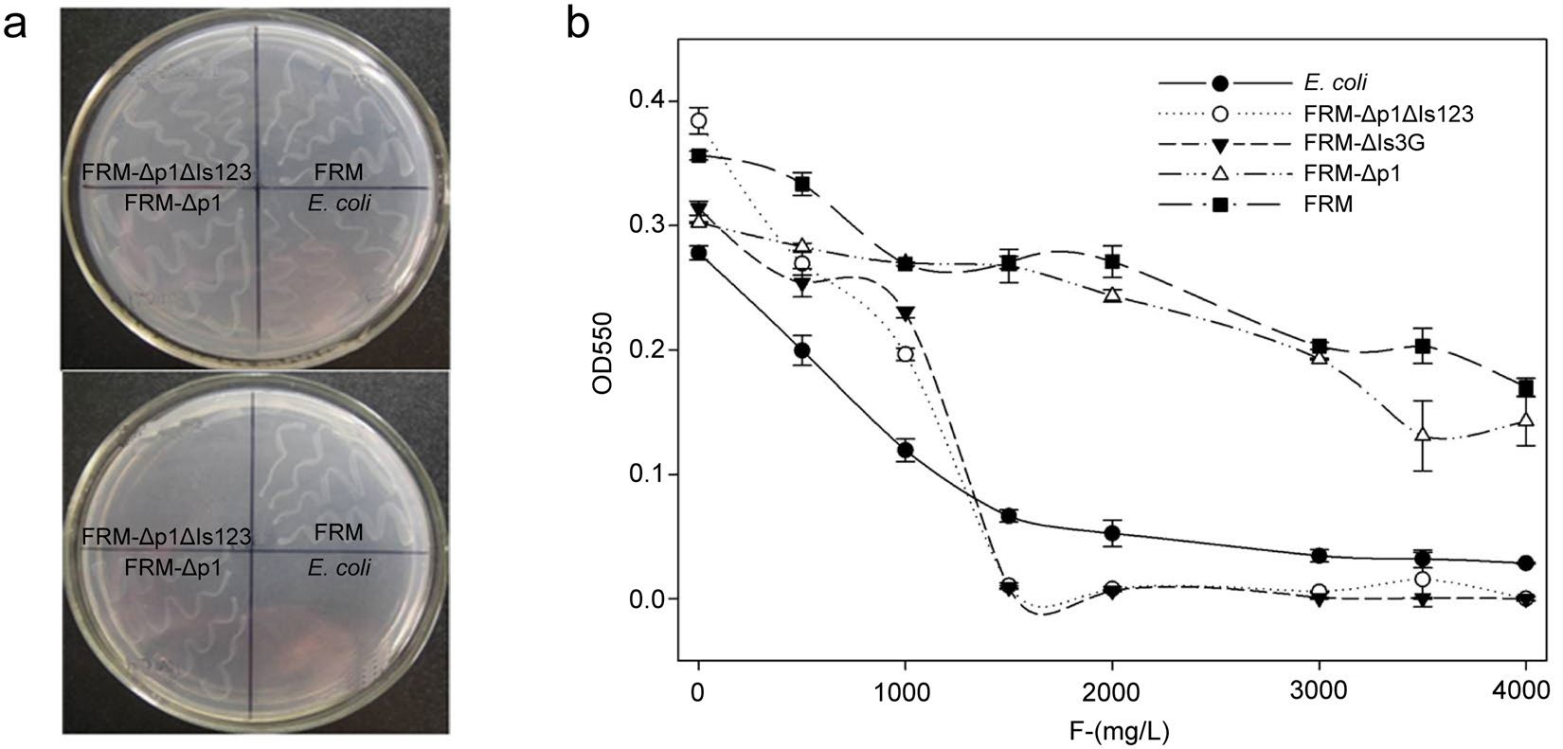
Examination of the fluoride resistance of *E*. *cloacae* FRM-Δp1ΔIs123 (elimination of plasmid p1 and the three genomic islands) and *E*. *cloacae* FRM-Δp1 (elimination of plasmid p1). (**a**) Four strains were streaked onto Burk plates without fluoride (upper) and with 1,000 mg/L fluoride (lower). (**b**) The minimum inhibitory concentration (MIC) of fluoride for *E*. *cloacae* FRM, *E*. *cloacae* FRM-Δp1ΔIs123, *E*. *cloacae* FRM-Δp1, and *E*. *cloacae* FRM-ΔIs3G.

To determine whether the genomic islands or plasmids contribute to the fluoride resistance of *E*. *cloacae* FRM, we eliminated the three genomic islands and the p1 plasmid. The resulting strains were verified by PCR using specific primers (Fig. S3) and their fluoride resistance was examined (Fig. 2). *E*. *cloacae* FRM-Δp1, which has lost the p1 plasmid, grew well on Bulk plates with and without fluoride, similar to the wild-type *E*. *cloacae* FRM strain, whereas *E*. *cloacae* FRM-Δp1ΔIs123, which has lost the p1 plasmid and the three genomic islands, could only be grown on Bulk plates without fluoride, similar to the *E*. *coli* control strain (Fig. 2a). In liquid media, the MIC of fluoride for both *E*. *cloacae* FRM-Δp1ΔIs123 and *E*. *coli* was 1,500 mg/L, while *E*. *cloacae* FRM-Δp1 grew well even at 4,000 mg/L fluoride, similar to *E*. *cloacae* FRM (Fig. 2b). These data indicated that the elimination of plasmid p1 does not influence the fluoride resistance of *E*. *cloacae* FRM. However, fluoride resistance was dramatically reduced when the three genomic islands were eliminated. Thus, we concluded that the genomic islands, rather than the p1 plasmid, contribute to the fluoride resistance of *E*. *cloacae* FRM.

The transcriptome of *E*. *cloacae* FRM following fluoride ion induction was analyzed by RNA sequencing. Reads per kilobase per million reads values were used to measure transcript abundance. As shown in Table 1, the expression level of some of the genes found on the third genomic island was greatly influenced by the presence of fluoride. For example, fluoride induction increased the expression of *crcB* (orf5251) 7-fold. The transcripts of the enolase gene *eno* (orf5255) and the universal stress protein gene *uspA* (orf5256) were increased 176- and 120-fold, respectively.

**Table 1:** Transcriptome annotation of the *Enterobacter cloacae* FRM genomic island GI3.

Gene	location		RPKM		Fold change	Annotation
	Start	End	untreated	induced		
orf05247	4068647	4069594	25.05	23.67	0.9	Efflux transporter, RND family, MFP subunit
orf05248	4069591	4072650	18.57	25.7	1.4	RND efflux transporter
orf05249	4072819	4073079	7.47	115.91	15.5	Divalent cation transporting ATPase
orf05251	4073189	4073449	20.47	166.01	8.1	Protein CrcB homolog
orf05252	4073598	4074080	28.62	281.65	9.8	2,3-bisphosphoglycerate-dependent phosphoglycerate mutase
orf05255	4074611	4075897	19.97	3533.52	176.9	Enolase
orf05256	4075913	4076341	15.02	1814.37	120.8	Universal stress protein A
orf05257	4076345	4077307	16.38	2721.34	166.1	Manganese-dependent inorganic pyrophosphatase
orf05258	4077805	4078017	156.12	280.39	1.8	Putative uncharacterized protein
orf05259	4078317	4078484	72.2	116.17	1.6	hypothetical protein

We also constructed a fosmid library from genomic DNA of *E*. *cloacae* FRM in *E*. *coli* and screened the clones with fluoride resistance to determine the genes that contribute to fluoride resistance. Among the total of 276 clones, only two clones, F1 and F2, could be grown on Bulk plates with fluoride at a concentration of 700 mg/L. The fosmid DNA isolated from F1 and F2 was sequenced. Sequence analyses of the two fosmids revealed that both contained the third genomic island, GI3.

**Figure 3:**
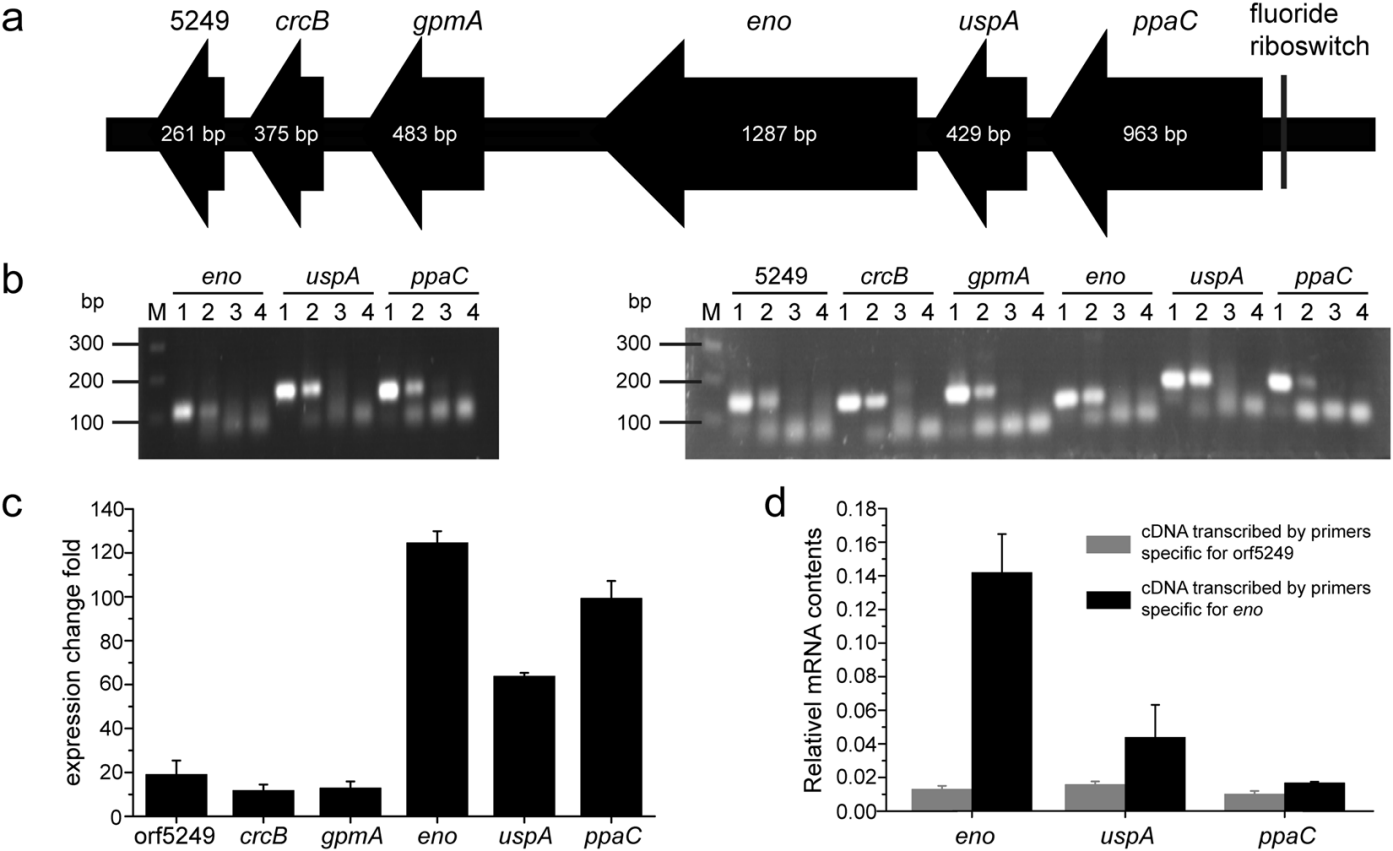
Genetic organization and expression of the Is3G fragment. (**a**) Schematic representation of the Is3G fragment loci. Thick black line, chromosomal DNA; filled arrows, six open reading frames with corresponding gene size; dotted lines, complementary DNA (cDNA) transcribed with gene-specific primers for orf5249 and *eno* in the 3′ direction. (**b**) Analyses of reverse transcription (RT)-PCR products (left, cDNA transcribed with gene-specific primers for orf5249; right, cDNA transcribed with gene-specific primers for *eno*). Lanes: M, 100-base-pair (bp) DNA ladder; 1, PCR positive control using *E*. *cloacae* FRM genomic DNA as a template; 2, RT-PCR using *E*. *cloacae* FRM cDNA as a template; 3, negative control using *E*. *cloacae* FRM RNA as a template; 4, PCR negative control without template. (**c**) Transcriptional analysis of the genes orf5249, *crcB*, *gpmA*, *eno*, *uspA*, and *ppaC* in *E*. *cloacae* FRM cultured with 1,000 mg/L fluoride in comparison with culture in the absence of fluoride using quantitative RT-PCR. Transcript levels of the tested genes were normalized to the 16 S rRNA gene. (**d**) The relative transcription levels of the genes *eno*, *uspA*, and *ppaC*. The 16 S rRNA gene was used as an internal control for normalization.

For further confirmation of the importance of GI3, the 4,489-bp GI3 fragment (designated as Is3G), which contains six genes (from orf5249 to orf5257, Fig. 3a) whose expression was increased by fluoride induction, was deleted and the fluoride resistance of this knockout strain, *E*. *cloacae* FRM-ΔIs3G, was evaluated. The fluoride MIC for *E*. *cloacae* FRM-ΔIs3G was 1,500 mg/L, the same as *E*. *cloacae* FRM-Δp1ΔIs123 and *E*. *coli* (Fig. 2b). Deletion of the Is3G fragment of *E*. *cloacae* FRM resulted in a loss of fluoride resistance. Our results suggested that the genomic island GI3, in particular the 4,489-bp Is3G fragment, is important for the fluoride resistance of *E*. *cloacae* FRM.

### The six Is3G genes are transcribed together and controlled by a fluoride riboswitch

To clarify the function of the indispensable Is3G fragment in fluoride resistance, the genetic composition and function of the genes located on this fragment were analyzed. We identified six genes on the Is3G fragment. The chromosomal organization of the six genes is shown in Fig. 3a and detailed annotation information is shown in Table 1. Some of the genes may be involved in fluoride resistance, such as *crcB*, which has been reported to encode a fluoride transporter protein that reduces the concentration of fluoride in cells^5^, and the gene *uspA*, which encodes the universal stress protein A that may be produced in response to a variety of stress conditions^13^. Two other gene products, enolase and pyrophosphatase, are essential for glycolytic metabolism and nucleic acid synthesis. These enzymes are inhibited by fluoride^14, 15^.

We scanned the Is3G fragment against the Rfam database^16^ to search for homologs of known RNAs and found a fluoride riboswitch (Rfam accession number RF01734) upstream of the gene *ppaC* (Fig. 3a), which regulates the expression of downstream genes^5^. This suggested that the expression of these six genes may be regulated by the riboswitch. To confirm this, we analyzed the expression of these genes. From the analysis of transcriptome, we found that the mRNA levels of the six genes were low in total RNA isolated from *E*. *cloacae* FRM cultured in the absence of fluoride. However, the mRNA levels were significantly increased in total RNA isolated from *E*. *cloacae* FRM cultured in the presence of fluoride. Fluoride induction increased expression of the genes *ppaC*, *uspA*, and *eno* by approximately 100-fold, whereas the expression of *gpmA*, *crcB*, and orf5249 was increased approximately 10-fold (Table 1). These results were confirmed by qRT-PCR (Fig. 3c). Given the large differences in expression induced by fluoride between the upstream and downstream genes, we hypothesized that *ppaC*, *uspA*, and *eno* are transcribed as one operon and *gpmA*, *crcB*, and orf5249 are transcribed as another operon. To confirm this, RT-PCR using total RNA extracted from *E*. *cloacae* FRM cultured in Burk medium with 1,000 mg/L fluoride was performed. The RT-PCR results, using an *eno*-specific antisense primer for reverse transcription, showed that *ppaC*, *uspA*, and *eno* are indeed co-transcribed (Fig. 3b, left). However, the genes *ppaC*, *uspA*, and *eno* were also detected in cDNA transcribed with an orf5249-specific antisense primer (Fig. 3b, right). This result suggested that the three downstream genes (*gpmA*, *crcB*, and orf5249) are also transcribed together with the three upstream genes (*ppaC*, *uspA*, and *eno*). However, the mRNA expression of the three upstream genes was greater than the expression of the downstream genes, which suggested that the mRNA of the upstream genes may not only be located on a single transcript. To verify this, qRT-PCR using cDNA transcribed with primers specific for orf5249 and the *eno* gene as the template was performed to compare the relative mRNA levels of the three upstream genes. The data showed that the levels of the three upstream genes in the cDNA transcribed with primers specific for the *eno* gene were greater than in the cDNA transcribed with primers specific for orf5249 (Fig. 3d). This suggested that there are two transcripts: one, which contains all six genes, and a second, which only contains the three upstream genes. Overall, these results indicated that the six consecutive genes reside in an operon and are transcribed into two mRNAs that are activated by fluoride through a fluoride riboswitch.

### An operon consisting of six genes confers high fluoride resistance

**Figure 4:**
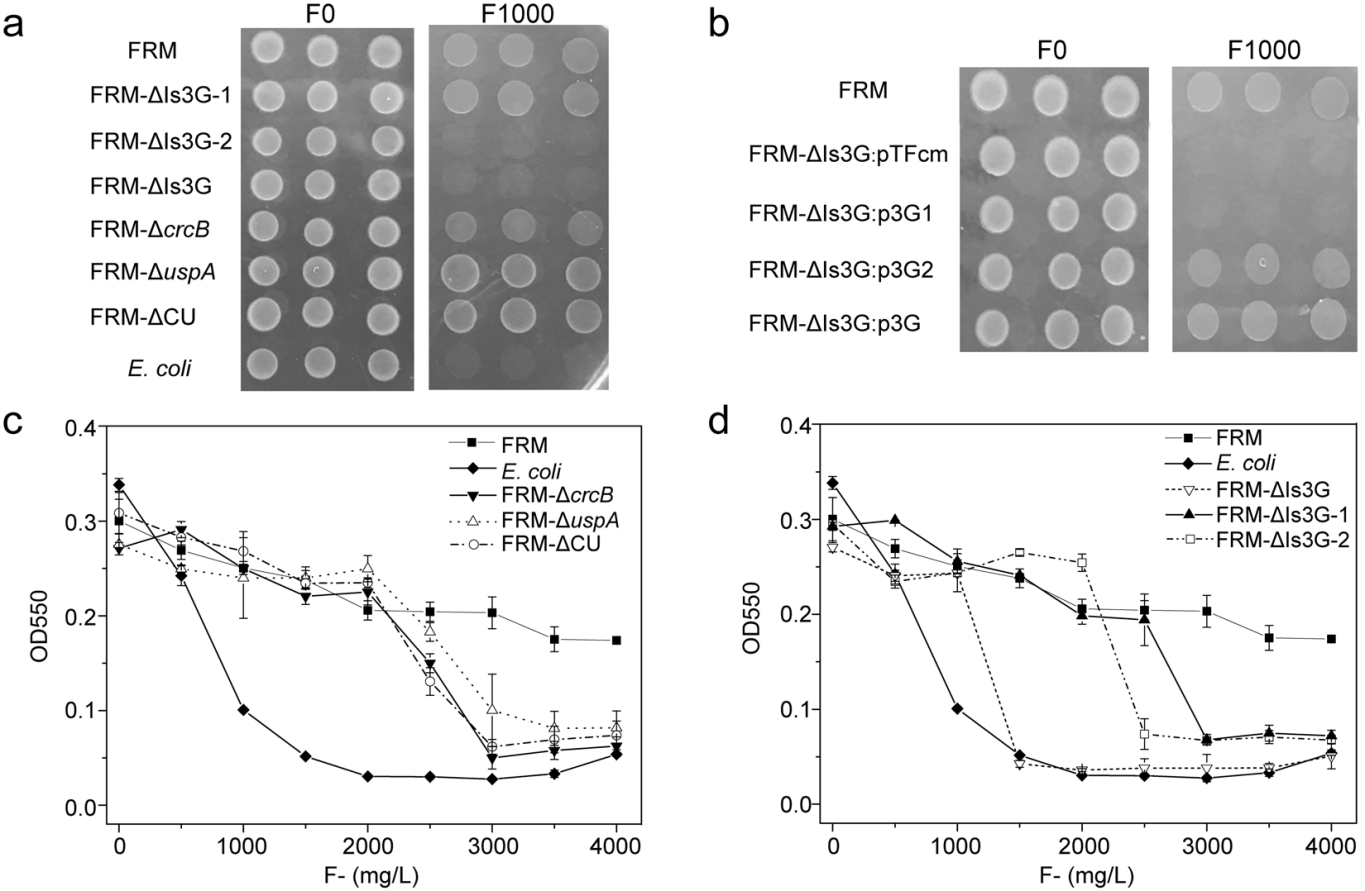
Examination of the fluoride resistance of *E*. *cloacae* FRM and knockout strains. (**a**) Eight strains were spotted onto Burk plates without fluoride (left) and with 1,000 mg/L fluoride (right). *E*. *cloacae* FRM-ΔIs3G: deletion of the Is3G fragment; *E*. *cloacae* FRM-ΔIs3G-1: deletion of orf5249, *crcB*, and *gpmA*; *E*. *cloacae* FRM-ΔIs3G-2: deletion of *eno*, *uspA*, and *ppaC*; *E*. *cloacae* FRM-Δ*crcB*: deletion of *crcB*; *E*. *cloacae* FRM-Δ*uspA*: deletion of *uspA*; *E*. *cloacae* FRM-ΔCU: deletion of *crcB* and *uspA*. (**b**) Rescue of fluoride toxicity by the fragments Is3G, Is3G-1, and Is3G-2 in *E*. *cloacae* FRM-ΔIs3G. (**c**) The MIC of fluoride for *E*. *cloacae* FRM, *E*. *cloacae* FRM-Δ*crcB*, *E*. *cloacae* FRM-Δ*uspA*, *E*. *cloacae* FRM-ΔCU, and *E*. *coli*. (**d**) The MIC of fluoride for *E*. *cloacae* FRM, *E*. *cloacae* FRM-ΔIs3G, *E*. *cloacae* FRM-ΔIs3G-1, *E*. *cloacae* FRM-ΔIs3G-2, and *E*. *coli*.

To determine the genes responsible for high fluoride resistance, we knocked out the genes in this operon to examine their contributions to fluoride resistance. As the *crcB* gene has been reported to be involved in fluoride resistance^5^ and the *uspA* gene is involved in physiological processes under conditions of stress, we first knocked out the *crcB* and *uspA* genes to determine their roles in *E*. *cloacae* FRM fluoride resistance. The knockout strains were verified by PCR (Fig. S4a and b). Deleting *crcB* and *uspA* did not cause a complete loss of fluoride resistance in *E*. *cloacae* FRM, as the deletion of *uspA* (*E*. *cloacae* FRM-Δ*uspA*), *crcB* (*E*. *cloacae* FRM-Δ*crcB*), and *uspA* and *crcB* together (*E*. *cloacae* FRM-ΔCU) still permitted growth on Bulk plates with 1,000 mg/L fluoride (Fig. 4a). The MIC for fluoride for all of these strains was 3,000 mg/L (Fig. 4c). These results suggested that there are additional genes involved in fluoride resistance.

Next, we deleted the fragment Is3G-1, which contains the genes orf5249, *crcB*, and *gpmA*, and the fragment Is3G-2, which contains the genes *eno*, *uspA* and *ppaC*, to make the knockout strains *E*. *cloacae* FRM-ΔIs3G-1 and *E*. *cloacae* FRM-ΔIs3G-2 respectively. These strains were verified by PCR (Fig. S4c) and their fluoride resistance was evaluated. Only *E*. *cloacae* FRM-ΔIs3G-2 and *E*. *cloacae* FRM-ΔIs3G could not be grown on Bulk plates with 1,000 mg/L fluoride (Fig. 4a). In liquid medium, only *E*. *cloacae* FRM-ΔIs3G showed weak fluoride resistance, similar to *E*. *coli*. The MIC for *E*. *cloacae* FRM-ΔIs3G-2 was 2,500 mg/L, lower than the MIC for *E*. *cloacae* FRM-ΔIs3G-1, Δ*uspA*, Δ*crcB*, and ΔCU (Fig. 4c and d). These results indicated that although deletion of these genes may influence fluoride resistance, only deletion of the whole Is3G fragment eliminates the high fluoride resistance of *E*. *cloacae* FRM.

To confirm the above results, we evaluated the recovery of fluoride resistance of the Is3G fragment. We transformed the plasmids containing the fragments Is3G, Is3G-1, and Is3G-2 into *E*. *cloacae* FRM-ΔIs3G to generate *E*. *cloacae* FRM-ΔIs3G:p3G, *E*. *cloacae* FRM-ΔIs3G:p3G1, and *E*. *cloacae* FRM-ΔIs3G:p3G2 respectively, and examined the fluoride resistance of these strains. As shown in Fig. 4b, the fragments Is3G and Is3G-2 allowed *E*. *cloacae* FRM-ΔIs3G to grow on Burk plates with 1,000 mg/L fluoride, similar to the wild-type *E*. *cloacae* FRM strain. Overall, these results indicated that all six genes found in the operon contribute to the high fluoride resistance in *E*. *cloacae* FRM.

## Discussion

Our research showed that an operon consisting of six genes is involved in the fluoride resistance of *E*. *cloacae* FRM. However, many microorganisms have been reported to use only fluoride transporters, such as the CrcB protein and a fluoride-specific subtype of chloride channels EriC^F^, to overcome fluoride toxicity^5, 7, 10–12^. These fluoride-resistant microorganisms do not survive extended exposure to 200 mM fluoride, indicating that the expression of fluoride channels alone is not sufficient to overcome this high concentration of fluoride. This was confirmed by our data: deletion of the *crcB* gene did not destroy the fluoride resistance of *E*. *cloacae* FRM. As *E*. *cloacae* FRM can tolerate a concentration of fluoride as high as 4,000 mg/L, and there are few microorganisms that can survive such a high concentration of fluoride, it was not surprising to find that *E*. *cloacae* FRM uses six different genes, including *crcB*, to overcome the toxicity of high fluoride levels.

We determined that the fluoride resistance-related operon was fluoride-induced and controlled by a fluoride riboswitch located on the promoter. The fluoride riboswitch and associated proteins have been reported as mitigation systems for fluoride toxicity that exist pervasively among bacteria and archaea^5^. The genes associated with the fluoride riboswitch have been analyzed by bioinformatics and found to encode proteins of diverse function^5, 6^. Our research provides the first experimental evidence linking additional fluoride riboswitch-associated genes to fluoride resistance. According to the function of these genes and their indispensable role in fluoride resistance, the mechanism of fluoride resistance for *E*. *cloacae* FRM could be clarified. Fluoride is detected by this riboswitch, which triggers the expression of the six genes that can help *E*. *cloacae* FRM to mitigate fluoride toxicity by expelling the anion from cells using the fluoride transporter (CrcB), by utilizing UspA and its role in universal stress adaptation, and by producing additional copies of enzymes (phosphoglycerate mutase, enolase, and pyrophosphatase) that are inhibited by fluoride.

The six genes of the fluoride resistance-related operon were transcribed into two separate transcripts. In addition to the transcript containing the six genes, we also identified a shorter transcript that contained only the three upstream genes, *ppaC*, *uspA*, and *eno*. This expression pattern revealed the precise effects that fluoride has on *E*. *cloacae* FRM. Additional transcripts of *ppaC* and *eno* suggested that fluoride may primarily influence glycolytic metabolism and nucleic acid synthesis by inhibited the key enzymes, enolase and pyrophosphatase, encoded by the *eno* and *ppaC* genes^14, 15^. The enzyme phosphoglycerate mutase, which is encoded by the *pgmA* gene, is also involved in glycolytic metabolism, but whether it is inhibited by fluoride has not yet been reported. Our data suggested that this enzyme may also be inhibited by fluoride, but this role may not be as important as the role of enolase in glycolytic metabolism.

Two transposases, orf5235 and orf5264, were also identified at the genomic island GI3, which are located upstream and downstream of the fluoride resistance operon, respectively. The identification of these transposases indicated that this unit of fluoride resistance was acquired by horizontal gene transfer (HGT)^17^. Thus, other bacteria could receive this genetic material through the same process. HGT may contribute to the spread of this fluoride resistance operon through the exchange of genetic material across genera, which will likely increase the number of fluoride-resistant bacteria.

Overall, our identification of an operon involved in fluoride resistance in *E*. *cloacae* FRM clarified the resistance mechanism and has expanded our understanding of the biological effects of fluoride.

## Materials and Methods

### Bacterial strains, plasmids, and growth conditions

The bacterial strains and plasmids used in this study are listed in Table S1. *E*. *cloacae* Agricultural Culture Collection of China (ACCC) 10453, *E*. *cloacae* ACCC 11690, *E*. *cloacae* ACCC 01620, and *E*. *cloacae* ACCC 11688 were purchased from the ACCC. *E*. *cloacae* ATCC 7256, *E*. *cloacae* CMCC 45301, and *E*. *cloacae* NCTC 9394 were purchased from China General Microbiological Culture Collection Center (CGMCC). *E*. *coli* Top 10 (TIANGEN Biotech, Beijing, China) was used as a cloning host. *E*. *coli* or *E*. *cloacae* were grown in lysogeny broth (LB) or modified Burk mineral medium [0.8 g/L KH_2_PO_4_, 0.2 g/L K_2_HPO_4_, 1 g/L (NH_4_)_2_SO_4_, 0.2 g/L MgSO_4_·7H_2_O, and 1 g/L yeast extract (Difco)] at 37 °C. NaF was used as fluoride. Ampicillin (100 mg/L), kanamycin (50 mg/L), or chloramphenicol (25 mg/L) was added as necessary.

### Evaluation of fluoride resistance

To evaluate the growth on plates with 1,000 mg/L fluoride, an overnight culture suspension was spotted or streaked on a plate and incubated at 37 °C for 24 h. To evaluate the growth in liquid medium, the minimum inhibitory concentration (MIC) of fluoride was determined. Burk broth (800 μL) with different concentrations of fluoride was dispensed into a 96-well (12 × 8) microtiter plate (96 × 1.5-mL wells) with a multi-channel micropipette (rows A to H: 0 mg/L, 500 mg/L, 1,000 mg/L, 1,500 mg/L, 2,000 mg/L, 3,000 mg/L, 3,500 mg/L, and 4,000 mg/L). Single colonies of the test strains were inoculated into 3 mL of LB broth and cultured overnight. A sample of this suspension (1%) was inoculated in 50 mL of LB broth and grown at 37 °C in an incubator at 200 rpm for 6 h. The test culture (15 μL) was then inoculated into each well of the prepared 96-well plate. After 20 h at 37 °C and 200 rpm, 200 μL of the cell suspension was transferred into a 96-well plate and the turbidity at OD_550_ was measured.

### Genome sequencing and analysis

Two Illumina libraries [0.5 kilobases (kb) and 3 kb] and Roche 454 paired-end (8 kb insert) whole shotgun libraries were constructed for *E*. *cloacae* FRM. Short reads generated from the Illumina paired-end library were assembled using Velvet 1.1^18^. Contigs were then joined into scaffolds with Illumina meta-pair reads and 454 paired-end reads. The gaps between the contigs were closed by PCR amplification and primer walking with an ABI 3730 sequencer. The transfer RNA genes were predicted with tRNAscan-SE^19^. The ribosomal RNA (rRNA) genes were identified with a basic local alignment search tool (BLAST) search against Rfam^16^ and rRNA gene sequences from TAC125. The open reading frames were identified with GLIMMER 3.0^20^. The predicted open reading frames were annotated by similarity searches against databases of nonredundant protein sequences from the National Center for Biotechnology Information, clusters of orthologous groups of proteins^21^, and InterPro^22^. The annotation of open reading frames was manually curated with Artemis^23^. The island in the genome of *E*. *cloacae* FRM was determined with the program IslandViewer 3^24^.

### Comparative genomics and phylogenetic analysis

The following genome sequences were used in genome comparisons with *E*. *cloacae* FRM: *E*. *cloacae* NCTC 9394 (accession number FP929040), *E*. *cloacae* SCF1 (accession number CP002272), *E*. *cloacae* ATCC 13047 (accession numbers CP001918, CP001919, and CP001920), *E*. *asburiae* LF7a (accession numbers CP003026, CP003027, and CP003028), *E*. *sp*. 638 (accession numbers CP000653 and CP000654), *E*. *aerogenes* KCTC 2190 (accession number CP002824), *Escherichia fergusonii* ATCC 35469 (accession number CU928158 CU928144), and *Escherichia coli str*. K–12 substr. MG1655 (accession number U00096). The sets of orthologous protein-coding genes were defined as mutual fully transitive reciprocal protein BLAST^25^ hits (with E-value < 10^−4^)^26^. Co-ortholog groups were identified by the reciprocal smallest distance (RSD)^27^. The nucleic acid sequence of each ortholog group was aligned using the CLUSTALW program, version 1.83^28^. The phylogenetic tree of the orthologous genes was constructed as a neighbor-joining (NJ) tree^29^ with the NEIGHBOR program from the PHYLIP package 3.67^30^. Bootstrap values were constructed using the CONSENSE program^30^ from 100 reproduced trees. The CONSENSE program was used to obtain the bootstrap values and generate the final phylogenetic tree.

### RNA sequencing and transcriptome analysis

An overnight culture of *E*. *cloacae* FRM was diluted 1:1,000 in Bulk medium in the presence (1,000 mg/L) and absence of fluoride, and the cultures were grown at 37 °C and 200 rpm to exponential phase (OD_550_ of approximately 0.6). Total RNA from both groups was extracted using TRIzol reagent (Invitrogen, Carlsbad, CA, USA) according to the manufacturer’s instructions. Isolated RNA samples were used for first-strand complementary DNA (cDNA) synthesis using random hexamers and Superscript II reverse transcriptase. Following end repair and the addition of a 3′-dA overhang, the cDNA was ligated to an Illumina paired-end adapter oligonucleotide mix and was size-selected for enrichment for 200-base-pair (bp) fragments by gel purification. After 16 PCR cycles, the libraries were sequenced using Illumina GAIIx. TopHat was used to map mRNA reads to the genome, and Cufflinks was used to calculate the expected fragments per kilobase of transcript per million mapped reads as expression values for each transcript.

### Elimination of plasmids and genetic islands

Plasmid and genetic island elimination was performed using sodium dodecyl sulfate (SDS, 0.6%) and a high temperature (42 °C) as previously described^31^. Single colonies were selected for on Burk plates without or with 1,000 mg/L fluoride and 100 μg/mL ampicillin to screen for the p1 plasmid and genetic island-eliminated strains. Primers p1-F and p1-R (Table S2) were used to identify the ampicillin-resistant gene located on the p1 plasmid. The primer pairs Is1-F and Is1-R, Is2-F and Is2-R, and Is3-F and Is3-R (Table S2) were used to detect the three genetic islands (Fig. S3).

### Construction and screening of an *E*. *cloacae* FRM genomic fosmid library

High-molecular-weight genomic DNA from *E*. *cloacae* FRM was extracted by the cetyl trimethylammonium bromide method^32^, and used to construct a fosmid library with the CopyControl Fosmid Library Production kit (vector pCC1FOS; Epicentre, Madison, WI, USA), according to the manufacturer’s instructions. A total of 276 clones were used to construct the library in three 96-well plates. To screen for clones with fluoride resistance, overnight cultures of the 276 clones were spotted onto Burk plates with 700 mg/L fluoride. fosmid DNA was isolated from the positive fosmid clones by alkaline lysis^33^ and sequenced from both ends on an ABI 3730 DNA analyzer with the pCC1 sequencing primers pCC1-F and pCC1-R (Table S2). The results were aligned with the genome sequence of *E*. *cloacae* FRM to confirm the sequence of the inserted fragments.

### Reverse transcription (RT)-polymerase chain reaction (PCR) and quantitative RT-PCR (qRT-PCR)

Total RNA was isolated as mentioned above in RNA sequencing. cDNA samples were prepared using TransScript One-Step gDNA Removal and cDNA Synthesis SuperMix (TransGen Biotech, Beijing, China) with gene-specific primers. An RT reaction using the primers 5249-RT and *eno*-RT (Table S2) as orf5249 and *eno*-specific antisense primers was performed, and the resulting cDNA was used as a template in PCR reactions for the detection of six genes with the following primers: 5249-F and 5249-R, *crcB*-F and *crcB*-R, *gpmA*-F and *gpmA*-R, *eno*-F and *eno*-R, *uspA*-F and *uspA*-R, and *ppaC*-F and *ppaC*-R (Table S2). qRT-PCR was performed using the SYBR Green Real-time PCR Master Mix Plus (Toyobo, Osaka, Japan) according to the manufacture’s instructions. 16 S rRNA (amplified with the primers 16SF and 16SR) was used as an internal control. All reactions were performed in biological triplicate, and the normalized fold changes of the relative expression ratio were quantified by the 2^−ΔΔCT^ method^34^.

### Construction of *E. cloacae* FRM knockout mutants

*E*. *cloacae* FRM knockout mutants were constructed by the CRISPR-Cas9 method, using the plasmids pCas (Addgene plasmid #62225) and pTargetF (Addgene plasmid #62226) as previously described^35^. Briefly, the chloramphenicol resistance (Cm^r^) gene (*cat*) amplified from pB1H1 with the primers CM-F and CM-R (Table S2) was inserted into the *Xho*I/*Mlu*I-digested pTargetF to replace the spectinamycin resistance gene (*aadA*), generating the plasmid pTFcm. To knockout the genes of interest, upstream and downstream fragments of each gene were amplified separately from *E*. *cloacae* FRM genomic DNA with the corresponding primers UF/UR and DF/DR (Table S2), and single guide RNA (sgRNA) containing a targeting N_20_ sequence of the loci of interest was amplified from pTFcm using corresponding SPF primers with the common reverse primer sgRNA-R. These three fragments were combined using an overlap PCR with SPF and DR primers, digested with *Spe*I/*Sal*I, and inserted into the *Spe*I/*Sal*I-digested pTFcm to construct the knockout plasmids (Table S1). These plasmids were then transformed into *E*. *cloacae* FRM harboring pCas and the deletion strains were screened as previously described^35^. Gene knockouts were verified by colony PCR with primers which were positioned ~600 bp upstream and downstream of the target genes (Fig. S4).

### Complementation

The fragments Is3G, Is3G-1, and Is3G-2 were PCR-amplified from *E*. *cloacae* FRM genomic DNA with the primer pairs 5249 L and 5257 R, 5249 L and 5252 R, and 5253 L and 5257 R (Table S2), and digested with *Hind*III and *Sac*I. These fragments were inserted into the *Hind*III/*Sac*I-digested pTFcm to create p3G, p3G1, and p3G2 (Table S1). The plasmids were verified by DNA sequencing and transformed into *E*. *cloacae* FRM-ΔIs3G.

### Data availability

The *E*. *cloacae* FRM chromosome and plasmid p1 and p2 sequences have been deposited in GenBank under accession numbers CP019889, CP019890, and CP019891.

## Electronic supplementary material

Supplementary Information
