# Supplementary material for: Identification of an operon involved in fluoride resistance in Enterobacter cloacae FRM
Source: Sci Rep. 2017 Jul 28;7:6786. doi: 10.1038/s41598-017-06988-1 (PMC5533749; doi:10.1038/s41598-017-06988-1)
Supplement: Supplementary file 1 — Supplementary Information [file 41598_2017_6988_MOESM1_ESM.pdf]

Identification of an operon involved in fluoride resistance in *Enterobacter cloacae* FRM

Xiaoqing Liu[1,+], Jian Tian[1,+], Lihui Liu[1], Tao Zhu[1], Xiaoxia Yu[1], Xiaoyu Chu[1], Bin Yao[2,*], Ningfeng Wu[1,*], and Yunliu Fan[1]

Supplementary Information

Table S1. Bacterial strains and plasmids used in this study.

Strains and plasmids	Description	Reference or Source
Strains		
E. coli Top 10	Clone strain	Our lab
Enterobacter cloacae		
FRM	Wild type	This study
FRM-Δ*crcB*	The *crcB* gene was deleted	This study
FRM-Δ*uspA*	The *uspA* gene was deleted	This study
FRM-ΔCU	Both the *crcB* and *uspA* gene were deleted	This study
FRM-ΔIs3G	The Is3G fragment containing orf5249, *crcB*, *gpmA*, *eno*, *uspA* and *ppaC* was deleted	This study
FRM-ΔIs3G-1	The Is3G-1 Isfragment containing orf5249, *crcB* and *gpmA* was deleted	This study
FRM-ΔIs3G-2	The Is3G-2 fragment containing *eno*, *uspA* and *ppaC* was deleted	This study
Plasmids		
pCas	*repA101*(Ts) *kan Pcas-cas9 ParaB-Red lacI*q *Ptrc*-sgRNA-*pMB1*	(35)
pTargetF		(35)
pB1H1	The template for amplication of the *cat* gene	Our lab
pTFcm	The *aadA* gene on pTargetF was replaced with the *cat* gene	This study
pTFcm-Δ*crcB*	sgRNA-*crcB*, 500-bp upstream and downstream of *crcB* were inserted into pTFcm	This study
pTFcm-Δ*uspA*	sgRNA-*uspA*, 500-bp upstream and downstream of *uspA* were inserted into pTFcm	This study
pTFcm-ΔIs3G	sgRNA-*ppaC*, 1000-bp upstream of *ppaC* and 1000-bp downstream of orf5249 were inserted into pTFcm	This study
pTFcm-ΔIs3G-1	sgRNA-*gpmA*, 1000-bp upstream of *gpmA* and 1000-bp downstream of orf5249 were inserted into pTFcm	This study
pTFcm-ΔIs3G-2	sgRNA-*ppaC*, 1000-bp upstream of *ppaC* and 1000-bp downstream of *eno* were inserted into pTFcm	This study
p3G	The Is3G fragment containing orf5249, *crcB*, *gpmA*, *eno*, *uspA* and *ppaC* were inserted into pTFcm	This study
p3G1	The Is3G-1 Isfragment containing orf5249, *crcB* and *gpmA* were inserted into pTFcm	This study
p3G2	The Is3G-2 fragment containing *eno*, *uspA* and *ppaC* were inserted into pTFcm	This study

Table S2. Primers used in this study.

Primer	Sequence (5'-3')[a]
Verification of the elimination of the plasmid p1 and the three genomic islands	
p1-F	TACGCTGGCCTTGGTGTCGTC
p1-R	GCGCTTGGATTTCCCTATGTC
Is1-F	CCAGCAGAACCAGTTAATTG
Is1-R	TGCGTACCACCTACTATAAG
Is2-F	CGCCGTATCAGTACCCATTG
Is2-R	ACGCCAGTAACGATTGTTAG
Is3-F	TGATTGATGAATTCGAAAGA
Is3-R	GGGCATCTCAGTGCTGACTA
pCC1 sequencing primers	
pCC1-F	GGATGTGCTGCAAGGCGATTAAGTTGG
pCC1-R	CTCGTATGTTGTGTGGAATTGTGAGC
RT-PCR and qRT-PCR	
5249-RT	GCGACTATCTGTGACCTCCGAATGA
eno-RT	TTTGAAGGCGGCTTTACCGGCGAA
5249-F	CGGACCAGGTGCGTGCTATC
5249-R	GGTGAATGGATGGTTCGTCGTTGT
crcB-F	ACTGTCCATGCGTCTCAATGC
crcB-R	TTCCAGGCGGGATCAAGGT
gpmA-F	CGTTTATCACTGGCGGAGGAG
gpmA-R	ACCTTCGTTGCGTCATTTCCA
eno-F	CCGTGCCTCCGTTCCTTC
eno-R	GACCGCATCCTGAACACCTT
uspA-F	CCGATGGTCTGCTGTTGCTTGA
uspA-R	GCGGGACTGACGGTCTTTGG
ppaC-F	CTGGTTGTGGCTGACTGGCTTA
ppaC-R	ACGGTGAGTAAATCGGGTTGTGAA
16S-F	CCGCATAACGTCGCAAGA
16S-R	AGTGTGGCTGGTCATCCT
Gene deletion	
CM-F	CGA**CTCGAG**AGCTTGATATCGAATTC
CM-R	TAT**ACGCGT**GATCCTCTAGAGCTTCGA
sgRNA-R	TCAAAAAAAGCACCGACTCGG
uspA-SPF	TCCTAGGTATAAT**ACTAGT**CTTGTACAGAATGAAACCGAGTTTTAGAGCTAGAAATAGC
uspA-UF	CGAGTCGGTGCTTTTTTTGAAGGCTGTGTTGCTGATGGGG
uspA-UR	AAATCACCTCTTATAAGAAG
uspA-DF	CTTCTTATAAGAGGTGATTTAAGAAGGTCAAACTTATGAG
uspA-DR	CCA**GTCGAC**GCGCCCTGCCAGCGGGCATGA
crcB-SPF	TCCTAGGTATAAT**ACTAGT**TCTTCCACCTGGTACCCTGGGTTTTAGAGCTAGAAATAGC
crcB-UF	CGAGTCGGTGCTTTTTTTGACAAAGATGAAGCCACTCAAC

crcB-UR	GGACAGTATCCAGCGGATCA
crcB-DF	TGATCCGCTGGATACTGTCCAAGCATATGCGCCATACACT
crcB-DR	CCA**GTCGAC**GATTGCAACCCGCGGATATG
Is3G-SPF	TCCTAGGTATAAT**ACTAGT**GATAATCCGATTTATGTCTTGTTTTAGAGCTAGAAATAGC
Is3G-UF	CGAGTCGGTGCTTTTTTTGACATAAACCCTCACTAATCCTGT
Is3G-UR	AGCTATACCTGACATACATGCC
Is3G-DF	GGCATGTATGTCAGGTATAGCTTCGCTGTCTCACACGCCGAATC
Is3G-DR	CCA**GTCGAC**CGTGACCACCCTGCGCGACCGT
Is3G2-DF	GGCATGTATGTCAGGTATAGCTCTTCTGATGCGTCACCTCCTTG
Is3G2-DR	CCA**GTCGAC**CATACCGATGATAAATCCGCCA
Is3G1-SPF	TCCTAGGTATAAT**ACTAGT**GGTTACCGGTGGACAAATCAGTTTTAGAGCTAGAAATAGC
Is3G1-UF	CGAGTCGGTGCTTTTTTTGAGGGAAGGGGTAGGGTGGTTC
Is3G1-UR	TATCAGCCAGAGCGAATGAAT
Is3G1-DF	ATTCATTCGCTCTGGCTGATATCGCTGTCTCACACGCCGAA
Is3G1-DR	CCA**GTCGAC**TACCGATGGGGTTTGTGGCG
Verification of gene knockouts	
uspA-yzF	CGGCGGATAAACCTTTTTCCAGCA
uspA-yzR	TCGGCATCATTGACCATCATCGTAT
crcB-yzF	TCGCTCTGGCTGATAATGGAA
ppaC-yzR	CCCGGGACTGTACTACCCTG
5249-yzF	TATCTGCCGATGGATGTTCTG
Complementation	
5249L	GCT**AAGCTT**CGAGTCCTGCCGTATCATCG
5252R	TGT**GAGCTC**CTTCTGATGCGTCACCTCCT
5257R	ATA**GAGCTC**CCCGGGACTGTACTACCCTG
5253L	CGC**AAGCTT**TATCAGCCAGAGCGAATGAA

[a] Restriction sites are indicated by bold characters. N_{20} sequences are underlined.

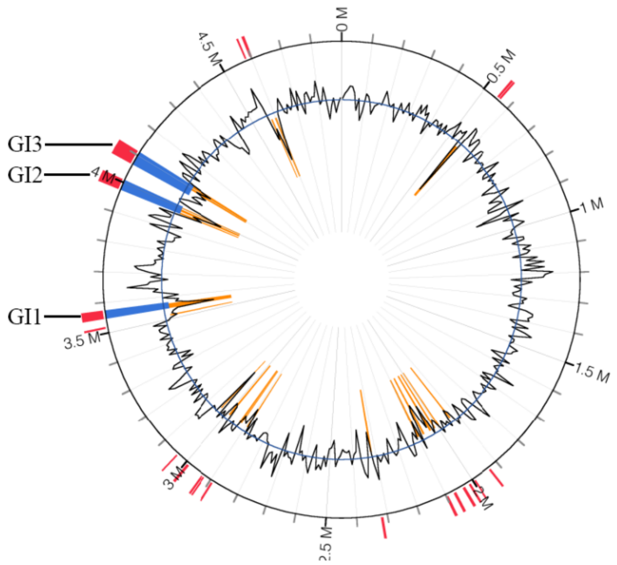

Figure S1. Analysis of genomic islands from *E. cloacae* FRM.

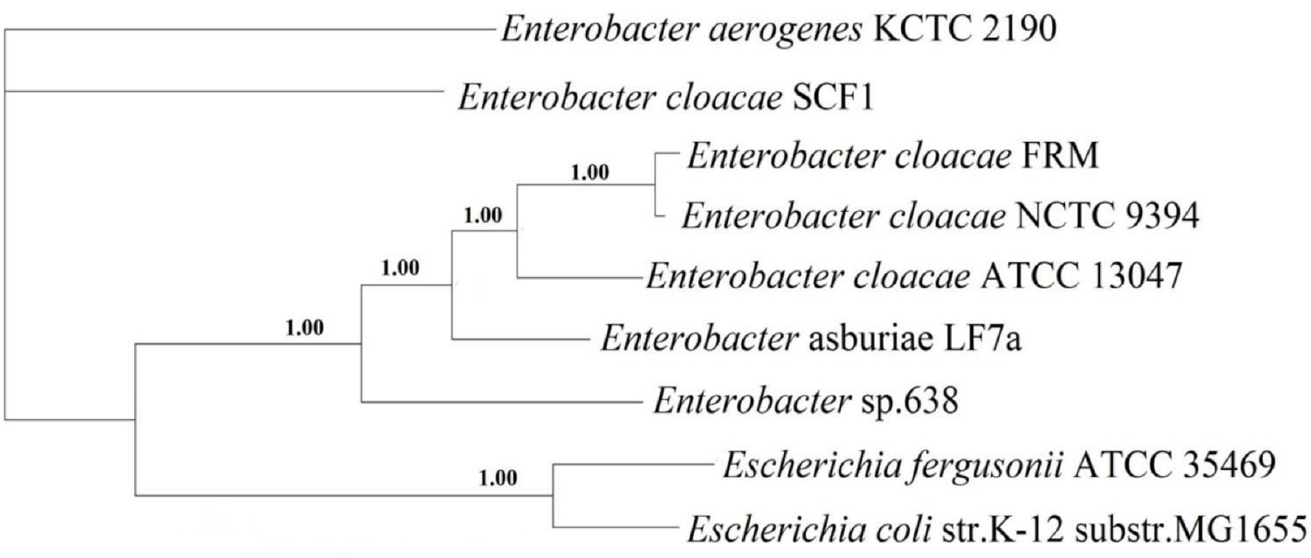

Figure S2. Genome-based *Enterobacteriaceae* phylogeny. Phylogenetic tree of seven different *Enterobacteriaceae* and two *Escherichia* strains was constructed from the sequences of 527 orthologous genes of the nine genomes, identified by the reciprocal smallest distance algorithm.

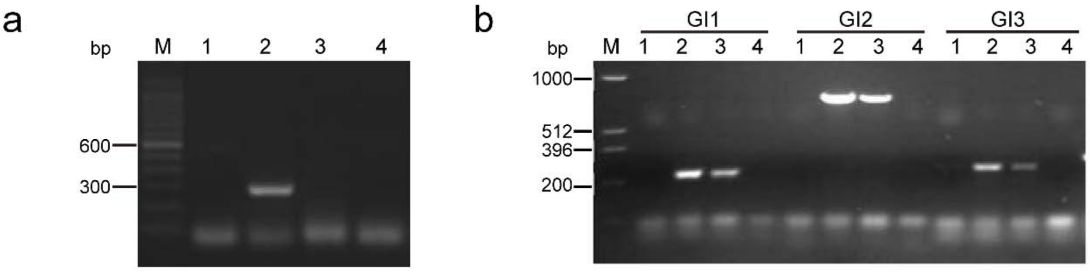

Figure S3. Verification of the elimination of the three genomic islands and the plasmid p1. (a) Identification of elimination of the plasmid p1 elimination by PCR with the primers p1-F and p1-R. Lanes: M, 100-bp DNA ladder; templates: 1, ddH_2O; 2, *E. cloacae* FRM genomic DNA; 3, *E. cloacae* FRM-Δp1ΔIs123 genomic DNA, and 4, *E. cloacae* FRM-Δp1 genomic DNA. **(b)** Identification of elimination of the three genomic islands (GI1, GI2, and GI3) by PCR with the primers Is1-F and Is1-R, Is2-F and Is2-R, and Is3-F and Is3-R. Lanes: M, 1-kb DNA ladder; templates: 1, *E. cloacae* FRM-Δp1ΔIs123 genomic DNA; 2, *E. cloacae* FRM-Δp1 genomic DNA; 3, *E. cloacae* FRM genomic DNA, and 4, ddH_2O.

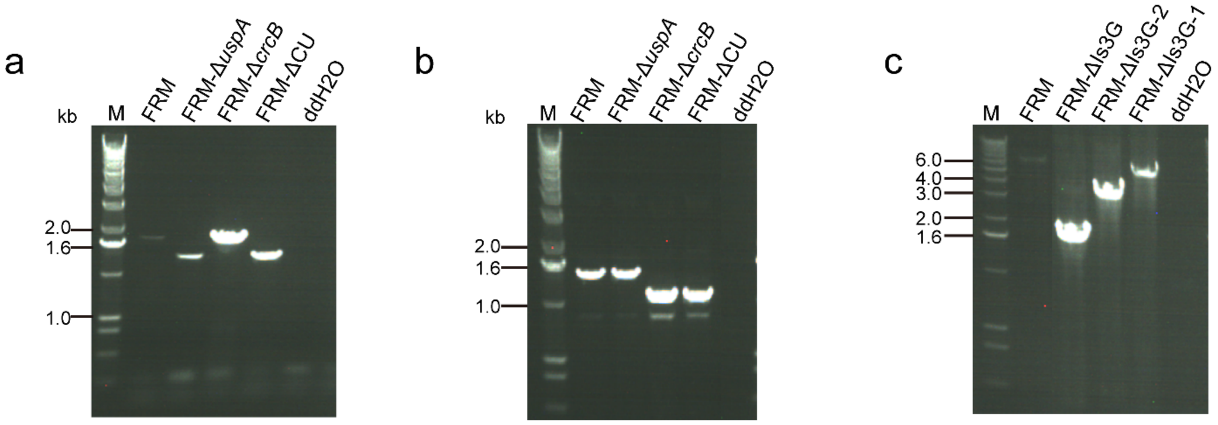

Figure S4. Verification of gene knockouts by colony PCR with primers (a) *uspA*-yzF and *uspA*-yzR located upstream and downstream of the *uspA* gene, **(b)** *crcB*-yzF and *crcB*-DR located upstream and downstream of the *crcB* gene, and **(c)** *ppaC*-yzR located upstream of the gene *ppaC* and 5249-yzF located downstream of orf5249.
